# Investigation of the Tribological Behaviour of Various AMC Surfaces against Brake Lining Material

**DOI:** 10.3390/ma16031001

**Published:** 2023-01-21

**Authors:** Sarah Johanna Hirsch, Patrick Eiselt, Ismail Ozdemir, Thomas Grund, Andreas Nestler, Thomas Lampke, Andreas Schubert

**Affiliations:** 1 Materials and Surface Engineering Group, Institute of Materials Science and Engineering, Chemnitz University of Technology, Erfenschlager Str. 73, 09125 Chemnitz, Germany; 2 Professorship Micromanufacturing Technology, Institute for Machine Tools and Production Processes, Chemnitz University of Technology, Reichenhainer Str. 70, 09126 Chemnitz, Germany

**Keywords:** aluminium matrix composites, brake disc, facing, friction, surface engineering, tribology, wear

## Abstract

AlSi7Mg/SiC_p_ aluminium matrix composites (AMCs) with a high ceramic content (35 vol.%) that were produced by using the field-assisted sintering technique (FAST) were subjected to tribological preconditioning and evaluated as a potential lightweight material to substitute grey cast iron brake discs. However, since an uncontrolled running-in process of the AMC surface can lead to severe wear and thus to failure of the friction system, AMC surfaces cannot be used directly after finishing and have to be preconditioned. A defined generation of a tribologically conditioned surface (tribosurface) is necessary, as was the aim in this study. To simulate tribological conditions in automotive brake systems, the prepared AMC samples were tested in a pin-on-disc configuration against conventional brake lining material under dry sliding conditions. The influence of the surface topography generated by face turning using different indexable inserts and feeds or an additional plasma electrolytic treatment was investigated at varied test pressures and sliding distances. The results showed that the coefficient of friction remained nearly constant when the set pressure was reached, whereas the initial topography of the samples studied by SEM varied substantially. A novel approach based on analysing the material ratio determined by 3D surface measurement was developed in order to obtain quantitative findings for industrial application.

## 1. Introduction

One of the major trends in the automotive sector is to use lightweight structural materials, such as high-strength aluminium alloys and steels. A lightweight material with high potential in both structural and wear applications is aluminium reinforced with hard particles [1,2,3,4]. Substituting conventional grey cast iron or steel components in friction systems, such as automotive brake discs, with aluminium matrix composite (AMC) parts could lead to a remarkable reduction in component weight while keeping the brake performance constant at least [5,6,7]. It was already revealed that AMCs have exhibited a profound improvement in tribological properties even in dry sliding condition that was linked to protective action of hard particles within the matrix alloy [8]. Additionally, various aspects, i.e., reinforcement shape and content, microstructure, matrix alloy, wear test configuration, applied load, sliding distance, etc., related to the wear behaviour of AMC were highlighted very well in the literature [9,10,11]. AMCs have also shown to offer great potential by providing improved wear performance compared to conventional materials. This is the case when the matrix alloy and the reinforcements are combined to a ratio where an increased load-bearing capacity is set and thus no severe deformation or fracture is expected during use. Furthermore, AMCs containing solid lubricant particles such as hexagonal boron nitride, graphite or molybdenum disulphide were also considered as favourable because of composite wear reduction [12,13,14]. The increase in resistance to wear of AMC was ascribed to intrinsic properties of solid lubricants, which enable it to form an effective lubricating layer with a low friction coefficient and wear loss. 

Despite many advantages of AMCs, the utilisation of wear-resistant aluminium composites that are reinforced with particles needs an adequate database, which could be directly transferred to their applications in automotive parts. For instance, the studies revealed that AMCs were suitable for use as brake discs [2,5,6,7,15,16], as they exhibited higher wear resistance than grey cast iron [15]. However, it is a fact that AMC brake discs require a special surface topography after machining, as the surface has a very strong influence on the wear of composite discs [17]. As a result, unfavourable surface conditions of the AMC components can lead to local frictional contact during the sliding movement between the brake lining material and the disc surface. High temperatures could develop at the contact areas, for example, leading to increased wear due to rapid failure of the protective tribologically conditioned layer (tribolayer) [12]. Therefore, the characteristic of this layer plays a critical role during the sliding of AMC brake disc surface against pin made of brake lining material. In other words, the formation of a stable tribosurface (tribologically conditioned) may act as a lubricant and decrease the wear rate. The fact is that the use of AMCs as a brake disc material in the automotive industry is still in its infancy. The presence of a particularly protective tribolayer formed during frictional contact was found to be strongly related to the local composition of the brake lining material [17]. The advantages of a continuous protective film formed between the composite and the lining during wear action are an improvement of wear protection and an effective reduction of disc noise emission. Most investigations on AMC and brake lining material pairing have evaluated wear parameters and they phenomenologically analysed the creation of the tribosurface [16]. In a review study, the potential use of AMC discs in the automotive industry was shown. It was summarised that AMC discs, which were also rubbed against conventional brake lining material, generally exhibit superior wear behaviour compared to grey cast iron. This behaviour was mainly attributed to the intrinsic properties of hard reinforcing particles when successfully incorporated into the matrix alloy during production [9]. On the other hand, it was claimed that machined AMC brake discs are not directly applicable in the automobile industry, as certain surface features require the formation of a protective tribolayer, which preserves AMC discs from excessive wear [18]. Additionally, our recent study indicated that the effective and reliable performance of AMC discs was strongly linked to the quick formation of a thicker and more stable tribolayer generated on the AMC contact surfaces [17].

The present study focuses on the role of surface preconditioning on the formation of a tribosurface during sliding wear of disc-shaped AMC samples tested against brake lining material. At first, field-assisted sintered disc-shaped AMC samples were prepared by facing or additional plasma electrolytical treatment to obtain three kinds of composite surfaces. Composite disc samples and brake lining pins were subjected to dry sliding wear tests at different loads and distances. The influence of preconditioned composite surfaces on the wear mechanisms was elucidated and then discussed in detail. 

## 2. Experimental Procedure and Methods

### 2.1. Material 

**Figure 1:**
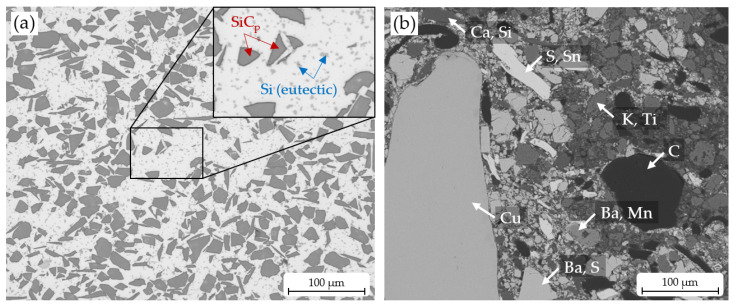
(**a**) The microstructure of a FAST sintered AMC reinforced with 35 vol.% SiC_p_ shows a regular distribution of particles, which are mainly responsible for the high wear resistance. The SiC_p_ (marked with red arrows) can be easily distinguished from eutectic silicon phase (marked with blue arrows) of the AlSi7Mg matrix alloy regarding their shape, size, and greyscale value. (**b**) A typical SEM image of the counterbody, a semi-metallic brake lining material, is shown with elements determined at points by EDX measurement. An EDX analysis of the whole area gave the following results (qualitative): Ba, Ca, Cu, K, Mg, Mn, S, Si, Sn, Ti, V, Zn, and Zr.

For the AMC production, gas-atomised AlSi7Mg cast alloy powder was selected as the matrix material, and 35 vol.% SiC particles (F400) were used as reinforcement for the aluminium composite. The mixed composite powder was consolidated by a field-assisted sintering process, in analogy to [19], to disc samples with a diameter of 200 mm and a height of 12 mm up to 14 mm. A typical microstructure of an AMC disc is represented in Figure 1a. It shows a relatively homogeneous distribution of SiC_p_ within the aluminium matrix structure. It can be seen that the reinforcing particles have an angular shape. Furthermore, there are spherical eutectic Si particles in the aluminium matrix alloy (light grey colour in the micrograph), which are shaped by FAST process (see [20]). 

To obtain an overview considering porosity, cross-sections were separated from three randomly chosen as-received AMC discs by abrasive water jet cutting and prepared in accordance with a common preparation routine. The average porosity was determined by digital picture analysis, evaluating greyscale values. The results are based on ten light microscope micrographs per cross-section with a size of (800 × 800) µm^2^ (microscope: Olympus GX 51, Evident Corporation, Tokyo, Japan; evaluation software: Olympus Stream Motion 2.1). The resulting porosity level was below the determination accuracy of the method of 1%. Therefore, the AMC material is considered to be almost pore-free. An average Brinell hardness of (99 ± 2) HB 62.5/2.5 was determined by five indents per cross-section. 

Abrasive water jet cutting was utilised to separate the AMC samples, considering an oversize of 1 mm in diameter. The radial distance of the samples to the centre of each as-received AMC disc was equal.

For the later counterbody, a common semi-metallic brake lining material was used. A scanning electron microscope (SEM) (ZEISS LEO 1455VP, Carl Zeiss Microscopy Deutschland GmbH, Oberkochen, Germany) image (Figure 1b) of a cross-section of this material shows the mixture of different phases. The respective elements are included within the image. An element analysis was conducted by energy dispersive X-ray spectroscopy (EDX).

### 2.2. Surface Preparation of Samples

The AMC samples were pre-turned to a diameter of 55 mm and a thickness of approximately 10.2 mm. Then machining tests were carried out with CVD diamond-tipped indexable inserts on a precision lathe (SPINNER PD 32, SPINNER Werkzeugmaschinen GmbH, Sauerlach, Germany) under dry condition. The tool radius and the feed were varied (Table 1), resulting in a different surface topography. 

For pin-on-disc tests, 18 samples of each surface condition were prepared. The samples in condition *E* were additionally plasma electrolytically treated (Beckmann Institut für Technologieentwicklung e. V., Germany) to remove the matrix alloy from the surface. For this purpose, each AMC sample was immersed in the electrolyte solution (watery acid solution, *ϑ* = 75 °C, *t* = 10 s) with the smoothly turned surfaces (see Table 1, condition *E*), facing downward. When the anodically switched sample comes into contact with the electrolyte solution, current flows abruptly, and a thin layer of gas or vapour forms between the sample and the electrolyte solution. It was given the name “plasma” because of its glow discharge. The process was very dynamic, meaning that the generation of new vapour or gas bubbles, their growth or unification, and their detachment from the sample toward the top occurred at a quick rate. After the process, the voltage was switched off, and each sample was taken from the solution, rinsed, and dried. 

**Figure 2:**
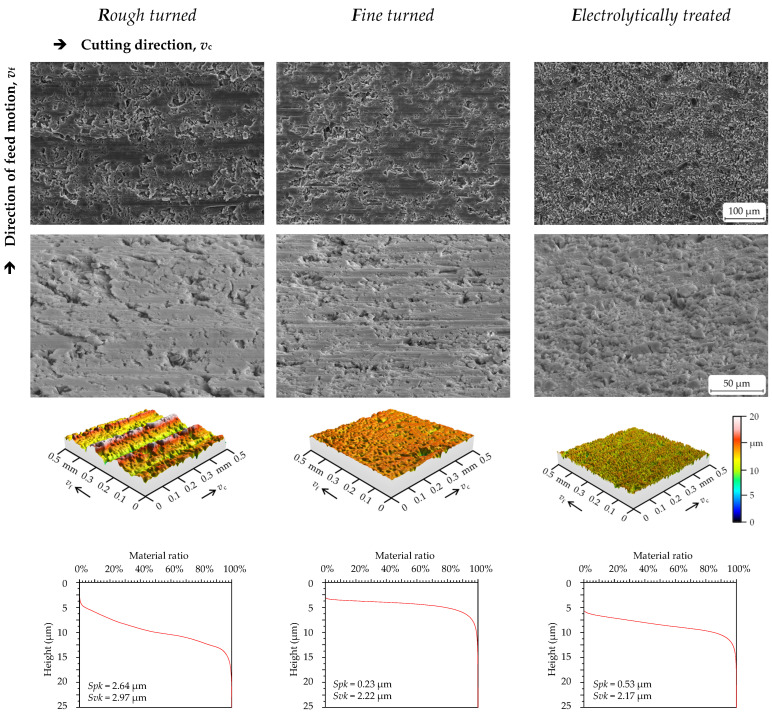
Secondary electron images taken by scanning electron microscopy (upper one taken vertically, and for bottom one, samples titled at an angle of 60° and higher magnification) show the surface topography of each surface condition. Below this, images of the 3D surface measurement (superelevated representation) and typical material ratio curves (MRCs, bottom) are shown.

The SEM, ZEISS LEO 1455VP, was used to characterise the microstructure and surface features of the AMC surfaces. Figure 2 depicts the typical surface appearance of machined (left and middle) and post-treated samples (right). The high porosity of the turned surfaces can be seen. Considering the SEM images of tilted samples with a wide view (Figure 2, second row), it seems that reinforcement particles are embedded beneath the sample surfaces after the turning process (left and middle), while SiC_p_ protrusions are clearly visible after plasma electrolytic treatment (surface *E*, right). Furthermore, the aluminium matrix material proportion on top of the surface was significantly reduced by the plasma electrolytic treatment, while SiC particles were more visible, indicating that the altered surface topography generated during plasma electrolytic treatment could enhance the formation of the tribosurface. Figure 2 also shows 3D surfaces and their corresponding material ratio curves (MRCs, also known as Abbott–Firestone curves; see Section 2.4). For the rough-turned samples (left), the kinematic roughness can be seen distinctly. The high roughness values are also reflected in the MRC, characterised by a strong slope and a largely reduced peak height (*Spk*). For the fine-turned surface, the kinematic roughness is only slightly pronounced, resulting in a decreased slope for the MRC and a significantly smaller *Spk* value. However, because of the high number and size of the pores, the reduced valley height (*Svk*) is much larger than the *Spk*. The electrolytically treated surface is characterised by protruding SiC particles involving a higher value for *Spk* in comparison to the fine-turned sample. Furthermore, there is a medium slope for the MRC.

### 2.3. Pin-on-Disc Test

Preconditioning tests were carried out using a pin-on-disc-type test bench (SRV, Optimol Instruments Prüftechnik GmbH, Munich, Germany) with an average pressure of 2 MPa or 5 MPa and a sliding velocity of 2 m∙s^−1^. After drying in an oven at 60 °C for 30 min, all samples were cleaned with ethanol and subjected to a pin-on-disc test. The counterpart pin with a diameter of 6 mm was made from common brake lining material. The detailed experimental setup is given in Table 2. As previously stated, the primary goal of this work was to determine whether the prepared surfaces (Table 1 and Figure 2) allow for a stable and rapid development of a tribosurface prior to reaching the steady-state friction regime. To accomplish this, analogous to [17], a pin was pushed against the AMC disc at an average radius of 21 mm while the disc rotated. To eliminate thermal expansion during testing, the samples were heated up to a temperature of 130 °C. Due to this, adjustment relevelling (AR) is mensurable as a change in height in the direction of the applied load. To avoid breakouts or even breakage of the pin, the load was gradually increased at the beginning of the test until an average contact pressure of 2 MPa or 5 MPa was reached. After 180 m of sliding distance, when the test pressure commenced to remain constant, the counting of the number of overruns started at 0*. 

Three samples were tested for each condition (combination of surface condition and test parameter set). For a first estimation of the wear behaviour in dependence of the different surface conditions, the mass of each AMC disc was determined before and after each pin-on-disc test by a precision balance (Sartorius MSA524S-100-DU, Sartorius AG, Goettingen, Germany). The same procedure was carried out with regard to the length measurement of the corresponding pins. An outside micrometre was used for this purpose.

The following equation and method were employed to compute the friction coefficients (*µ*) and AR rate, using the raw test data of *F*_f_ (friction force) and *F*_N_ (normal load):

(1) $$\mu =\frac{F_{f}}{F_{N}}$$

For all test distances, the AR rates were determined for sections I (0*–500 overruns), II (500–2000 overruns), and III (2000–10,000 overruns). The AR rate was defined as the slope of a straight line created by linear regression in sections I to III of the AR curves.

It should be noted that, prior to subjecting the plasma electrolytically treated samples to the pin-on-disc test, an additional extraction system with a high vacuum and compressed air was installed to reduce severe wear of pin and disc due to the three-body friction paring (action). 

### 2.4. 3D Surface Measurement and New Evaluation Method

The surfaces were captured by using a 3D laser scanning microscope (VK-9700, Keyence Corporation, Osaka, Japan). For each sample, one stitched measuring area was detected with a 50× objective at the same position before and after the pin-on-disc-tests. The analysed measurement field had a size of 2 mm × 2 mm. 

A surface analysis software (MountainsMap 7.4, Digital Surf, Besançon, France) was utilised for the evaluation of the surfaces applying different mathematical operations. After aligning the surface, a 5th degree polynomial was used to remove shape deviations. Subsequently, because of the porous surface topography, a robust Gaussian filter was applied to eliminate the waviness of the surface. The nesting index of the robust Gaussian filter was selected according to DIN EN ISO 25178-3 and amounted to 0.25 mm. Special attention was focused on the MRC. Before the pin-on-disc test, the reduced peak height (*Spk*) and the reduced valley height (*Svk*) were determined.

**Figure 3:**
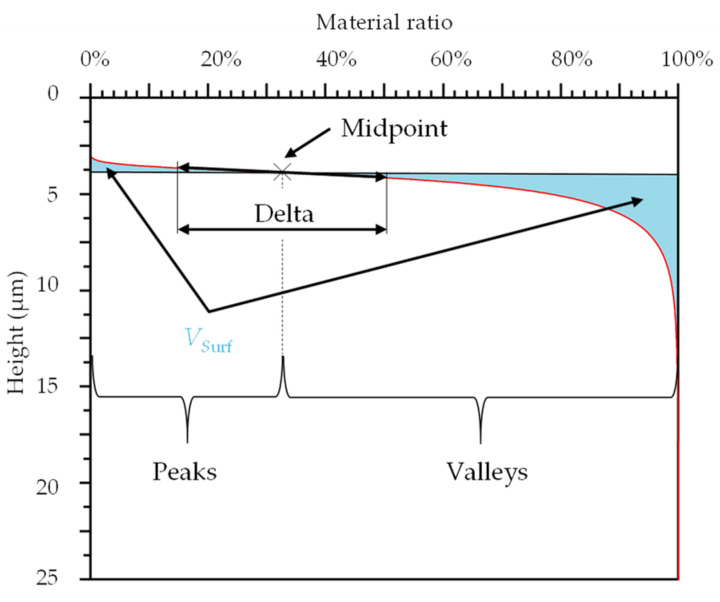
The picture represents the new surface parameter, *V*_Surf_. As an example, an MRC for a face-turned surface before the pin-on-disc test is shown. To determine the *V*_Surf_, the areas below and above the MRC (light blue colour) are calculated by integration and added.

For the calculation of the volume fractions (new parameter, *V*_Surf_; Figure 3), the datapoints of the MountainsMap MRC were imported to a special MATLAB programme (MATLAB, MathWorks, Natick, MA, USA). 

For the determination of the *V*_Surf_, the almost-linear range of the MRC was approximated by a straight line. A coefficient of determination (*R*^2^) with a value of 0.997 was used to calculate delta. The height of the intersection point of a vertical straight line in the middle of delta and the MRC was applied to separate the material surface into peak and valley areas. The size of the two areas was determined mathematically via integral functions. The parameter *V*_Surf_ is the sum of both areas. The calculation of *V*_Surf_ was carried out for each sample before and after the pin-on-disc test.

### 2.5. Microstructural Analysis of AMC surface

SEM was used to characterise tribologically conditioned surfaces of AMC discs based on the topography (SE, secondary electrons). SE images were taken before and after the pin-on-disc test to investigate the tribosurface formation for each parameter setting.

In addition to the top-view SE images of tribosurfaces, SE images of tilted samples (with an angle of 60°) were taken to assess the preconditioning behaviour of the samples. Furthermore, EDX mappings were recorded by SEM (ZEISS NEON40EsB, Carl Zeiss Microscopy Deutschland GmbH, Oberkochen, Germany) to evaluate the formation of the tribosurface, i.e., to assess where typical elements transferred from the counterbody (e.g., Cu and Ba) were found in addition to the expected elements of a reactive tribolayer for the AMC matrix material, i.e., Al, Si, and O.

## 3. Results 

### 3.1. Friction and Wear Behaviour

**Figure 4:**
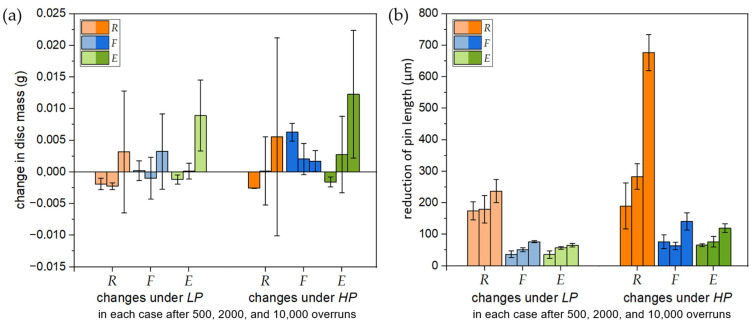
(**a**) The diagram shows the changes in disc mass for every surface condition and test pressure after 500, 2000, and 10,000 overruns. No significant mass changes until 2000 overruns are detectable, except for surface *F* tested under *HP*. While the surfaces *R* (under *LP* + *HP*) and *F* (under *LP*) showed almost no mass changes, a significant mass increase of surface *E* samples was obtained for *LP*, as well as for *HP* after 2000 overruns. (**b**) The average values of the pin-length reduction for the investigated surface conditions and preset pressures show that higher pressure leads, in general, to a larger pin-length reduction. In addition to that, surfaces *F* and *E* entail a lower pin-length reduction combined with a smaller standard deviation in comparison to surface *R*.

Changes in the mass of the AMC discs and the pin length were determined as described in Section 2.3. Figure 4a shows the mass difference for all surface and test conditions excluding samples that were severely worn due to damaged pin material. It can be seen that mass difference of samples tested with short and medium distance varies and is close to zero. 

After 10,000 overruns (1500 m sliding distance in total), the mass seemed to rise a little bit in on average, but with a high standard deviation. The length loss of pins rubbing against surfaces *R*, *F*, and *E*, as shown in Figure 4b, exhibited a different behaviour. 

It is obvious that the reduction of the pin length rubbing against *R* samples differs by a factor of around five compared to the *F* and *E* samples, and the standard deviation is higher, as well. The diagram additionally indicates that, with 5 MPa (*HP*), the pin loss increases for all surfaces (see Figure 4). This is consistent with the literature’s finding that the wear loss rises with the load and sliding distance or duration [7,15,21]. 

**Figure 5:**
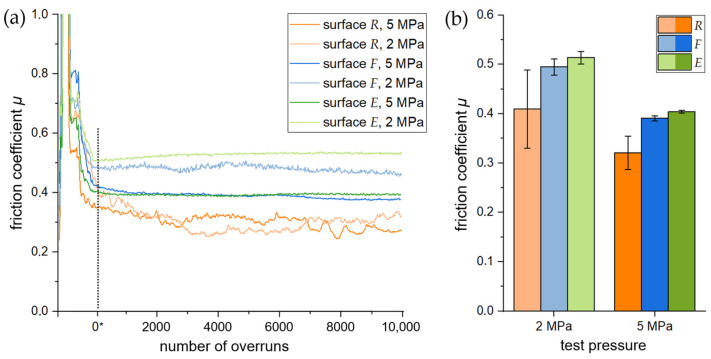
(**a**) Typical curves of the friction coefficient (*µ*) until 10,000 overruns (1500 m sliding distance) are shown for both test pressures and each surface condition. When preset test pressure is achieved (at 0* overruns), *µ* does not change longer. (**b**) It can be seen that *HP* (5 MPa) leads to a lower *µ*. While surfaces *F* and *E* show a similar behaviour, surface *R* is characterised by lower friction coefficients with higher standard deviations.

During the test, the *F*_f_ and AR in the direction of the pin axis were recorded to ensure a constant average test pressure. Typical curves of the friction coefficient (*µ*) that were calculated by using Equation (1) are shown in Figure 5a. The curves of the long tests were selected to provide an overview of the tribological behaviour over a high distance (1500 m in total; see Table 2). The first noticeable thing to point out is that *µ*-curves do not change significantly once the test pressure is set (0*, see Figure 5a). While the curves in Figure 5a provide a qualitative impression of the friction behaviour of the surfaces, the diagram in Figure 5b shows the averaged values of all investigated surfaces calculated from the beginning (0*) until the end of the test. 

As can be seen in Figure 5, surfaces *F* and *E* show similar behaviour: at *HP* (dark lines) friction coefficient is smaller compared to tests with *LP* (light lines). Both *µ*-curves of surface *R* at *LP* and *HP* run below those of surfaces *F* and *E* and do not show a significant difference. In addition, it can be noted that the *µ* (Figure 5b) of surfaces *E* and *F* was about 0.5 for *LP* and 0.4 for *HP*, while that of surface *R* was approx. 20% lower on average for all conditions. In general, the difference between *HP* and *LP* was almost the same for all surfaces with −20% in the middle. However, surface *R* stands out again with a standard deviation four to seven times higher compared to the other surface conditions (see Figure 5b). 

**Figure 6:**
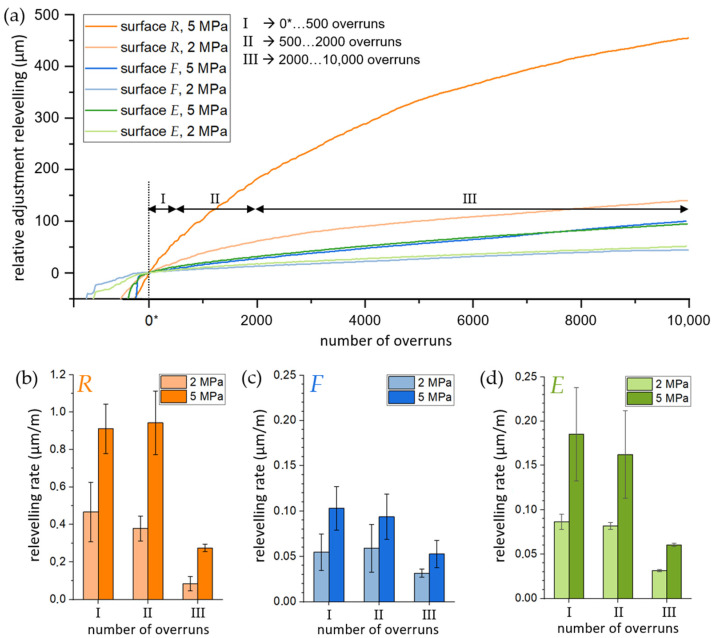
(**a**) Typical AR curves of the long tests (1500 m sliding distance) for each surface condition and preset pressure. AR rate (**b**–**d**) is determined after reaching constant test conditions (0* overruns). Note that scale of (**b**) differs from that of (**c**,**d**).

To examine the changes in AR more comprehensively, Figure 6a shows the changes plotted against increasing number of overruns. In addition to that, Figure 6b–d represent the rate of AR in sections of 0* to 500 overruns, 500 to 2000 overruns, and 2000 to 10,000 overruns, sequentially calculated based on the description in Section 2.3.

The curves indicate a significantly higher AR for surface *R* than for samples *F* and *E*. This is confirmed by the AR rate. For all sliding distances (I, II, and III; see Figure 6), surface *R* led to a higher rate on average (see Figure 6b) compared to surfaces *F* (Figure 6c) and *E* (Figure 6d). At both low and high pressures, the AR rates for surface *R* were obviously ten and six times higher than that for surfaces *F* and *E*, respectively. Considering the calculated mean values, the AR rate increased by approx. 100% for the *R* sample and by approx. 80% for the *F* and *E* samples when the test pressure was increased from *LP* to *HP*. Furthermore, the lowest AR rate was reported for all samples calculated from 2000 to 10,000 overruns. Nonetheless, in *LP* and *HP* conditions, the AR rate of surface *R* is two to five times higher than those of surfaces *F* and *E*. It is important to note that certain trends might be noticed when looking at shorter test periods (0*–500 overruns and 500–2000 overruns): While surfaces *F* and *E* showed no significant changes under *LP*, the *R* sample’s AR rate decreased noticeable. In contrast, a slight decrease in the average AR rate under *HP* was observed for surfaces *F* and *E*, while the rate for surface *R* remained at a high level. 

### 3.2. Microstructural Observations

**Figure 7:**
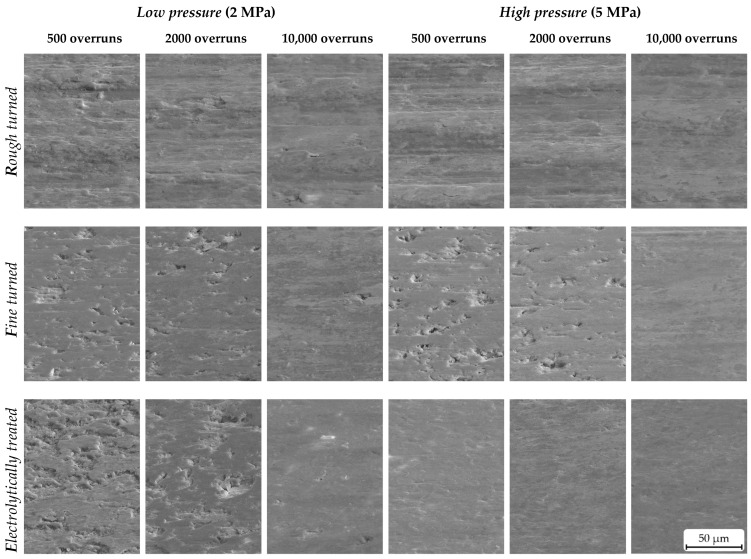
SE images taken from samples tilted at an angle of 60° qualitatively show the influence of the surface treatment and the test parameters on the formation of the tribosurface. While surface *R* shows a smooth surface for *LP* and *HP* already after a short sliding distance (500 overruns), surface *F* does not seem to be prepared before 2000 overruns for *LP* and *HP*. For the surface *E*, a similar behaviour can be seen for *LP*, but under *HP*, the surface is already levelled after a short sliding distance.

Figure 7 shows typical topographical changes in the tribosurfaces resulting from different load for short, medium, and long sliding distances. For all surfaces and test conditions, the selected SE images of tilted samples show that, as the test duration increases, the surface becomes smoother, as was rarely observed in [21]. Under the test condition with *HP*, it appeared that surfaces *R* and *E* reached a flat and closed tribosurface after only 500 overruns. Lee at al. [22] observed a similar behaviour: with increasing pressure, more noticeable smoothening occurs. This behaviour also was observed for surface *R* under *LP*, whereby slight differences in the height can still be observed after 500 overruns. Compared to the surfaces *R* and *E* (under *HP*), surface *F*, as well as this of condition *E* (under *LP*), does not appear to be completely dense after 2000 overruns. Surface imperfections, such as cavities caused by particles removed during turning, were not completely levelled.

**Figure 8:**
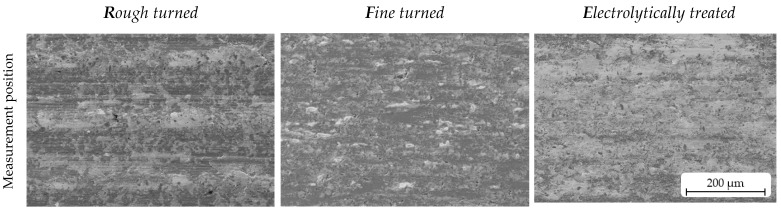

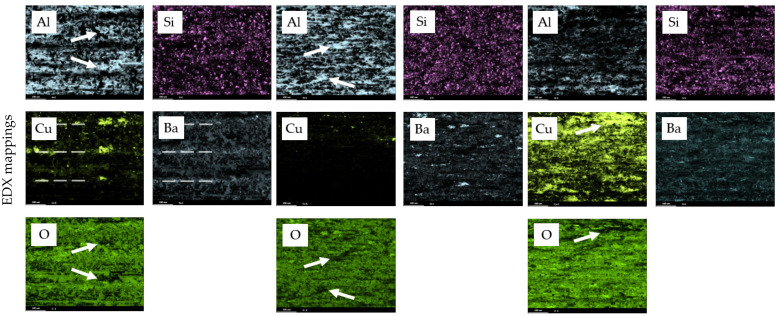
Typical SE images taken from wear tracks of surface *R* (**left**), *F* (**middle**), and *E* (**right**) after medium distance under *HP*. It can be seen that tribosurface builds up in different ways (compare EDX mappings in the second row).

In addition to the SE images of the tilted samples (Figure 7), EDX mappings were recorded of all surface conditions after a medium test distance at *HP* (Figure 8). In all cases, material transfer can be observed. The investigations of Lee et al. [22] presented that the minimum pressure for tribosurface formation was approx. 1.5 MPa. The element Al belongs to AMC disc material, as well as the majority of the detected Si (eutectical silicon and SiC_p_). Cu and Ba are selected representative elements of the counterbody material. As can be seen for all surfaces, a high concentration of Cu and Ba lowers the relative concentration of Al and Si. In addition to that, all three surfaces show finely dispersed oxygen.

The different surface types, however, present specific features. In the case of surface *R*, Cu was mainly transferred as bigger patches at the former ridges of the turning marks (indicated with dashed lines). In comparison, the investigated region of surface *F* seems to have nearly no transferred Cu. This may be attributed to the inhomogeneity and size of Cu included in the counterbody material (brake lining, see Figure 1). Depending on the location of bigger Cu particles in the pin material, more or less Cu was transferred to the AMC surface. Barium-containing phases, on the other hand, are smaller and better distributed in the brake lining material (see Figure 1), so that a continuous transfer over the whole tribo-track can be expected. The EDX mappings confirm this. An equally distributed counts for Ba can be seen, but in different appearances with respect to the AMC surface characteristics. While the transferred Ba in surface *R* looks like a network with higher concentration at the former turning marks (comparable to Cu), more or less irregular distributed “spots” of Ba are filled in places assumed to be former pores of surface *F*, which can be seen more clearly in Figure 7. 

The tested surface *E* shows a mixture of both a network and spots. In addition to that, the pin-material transfer on surface *E* seems to have occurred more uniformly. This may influence the later application behaviour, since the uniformity and thickness of tribosurfaces were determined to have a substantial impact in delaying the mild-to-severe wear of AMC [12]. In comparison with the other elements, oxygen shows, in all cases, a regular distribution over the whole SEM image. This confirms a reactively generated, i.e., oxidised, tribolayer that is homogeneously formed within the whole area of body/body contact during the tribological treatment. A few regions with less counts for oxygen correlated with Al (for surface *R*, *F*, and *E*) and Cu (for surface *E*) (see arrows in Figure 8).

In addition to the EDX mapping, EDX measurements for elemental identification were carried out. The detected elements are those that were determined in the pin material (see Figure 1b): Ba, Ca, Cu, K, Mg, Mn, S, Si, Sn, Ti, V, Zn, and Zr. 

### 3.3. Surface Volume

**Figure 9:**
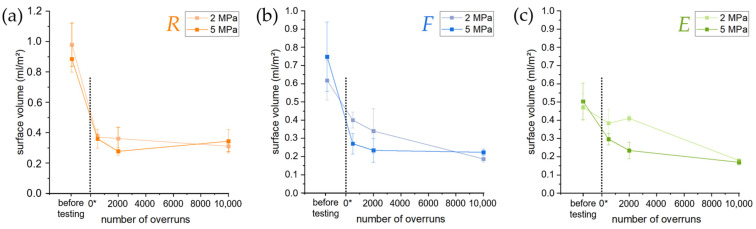
The surface volume as new surface characteristic shows changes with the increasing number of overruns. While the surface *R* (**a**) indicates no significant difference between preconditioned surfaces tested under *LP* and *HP*, surfaces *F* (**b**) and *E* (**c**) show smaller *V*_Surf_ values after testing with *HP*. For surface *R*, no clear changes in surface volume are observed after 0*. For surfaces *F* and *E*, the surface volume slightly decreased with the increasing number of overruns. Note that the scale of (**a**) differs from that of (**b**,**c**).

In addition to the friction coefficient, as a typical tribological parameter, the new surface characteristic, *V*_Surf_ (surface volume), was determined, and the results are shown in Figure 9. The values of *V*_Surf_ before preconditioning were also added to the chart in order to compare the surface volume of samples evaluated after a short, medium, and long sliding distance. While the *V*_Surf_ of the preconditioned surface *R* fluctuates for both pressures around 0.35 mL/m^2^ without any trend, the *V*_Surf_ values of the surfaces *F* and *E* decreases with the increasing number of overruns. A similar trend was found for an increasing pressure up to 2000 overruns. However, after 10,000 overruns, the difference was visible in the lowest values obtained, which amounted to 0.20 mL/m^2^ for surface *F* and 0.18 mL/m^2^ for surface *E*. Compared to the pre-test condition, the *V*_Surf_ decreases by approx. two-thirds for surface *R*, while it decreased by about half and one-fourth for surfaces *F* and *E*, respectively, when tested up to 500 overruns. 

## 4. Discussion 

The change of the AMC disc mass and decrease of the pin height were both examined for different tribological setups. The investigated variation of disc surface condition, pin pressure, and number of overruns did not strongly affect the resulting mass of the AMC discs. The increased wear resistance of AMC in comparison to the non-reinforced matrix material was mostly ascribed to a strengthening of the matrix by hard-particle reinforcement. In addition, it was related to the formation of a protective tribosurface and the superficial SiC_p_ load-bearing capability [21,23]. However, under severe wear conditions, single SiC particles are prone to fracture, hence lowering the load bearing capacity and thereby leading to the removal of single SiC particles, as well as matrix cracking, which are common failure stages in AMC reinforced with low particle volume fractions (up to 20 vol.%) [22,24]. In this study, the results showed that no severe wear of the AMC body occurred, even at *HP* (see Figure 4). This indicates that the load-bearing capacity prolonged for all the surfaces and test conditions. This is attributed to AMC reinforced with high volume fractions, as described in [22] for AMC with 55 vol.% SiC_p_. 

However, a partially strong reduction in the pin height was reported for all setups. The pin material was removed especially severely during the first 2000 overruns on rough-turned AMC surfaces (*R*), indicating a significant change in the surface characteristics. The corresponding SE images (Figure 7) confirm a smooth transfer or tribologically conditioned reaction layer already at the early stages (up to 2000 overruns) of sliding contact between the pin and disc. This tribosurface is obviously not formed or in fragmentary state at fine-turned (*F*) and electrolytically treated (*E*) AMC surfaces at equal numbers of pin overruns. The adjustment relevelling (AR) rates (Figure 6) confirm this observation. Surface *R* shows a constantly high AR rate level, i.e., pin wear, up to 2000 overruns. Both surfaces *F* and *E* generally indicate lower AR levels and a more distinctive AR drop towards higher numbers of overruns. The very high AR level of *R* samples in comparison to the other sample types is interpreted as (ongoing) tribological layer removal and formation under the high shear deformation of the counterbody transfer. Such behaviour is restricted to areas of direct contact between two wear parts, resulting in an increase in the AR (or wear) rate [6].

With respect to abrasion, AMC exhibited better wear performance against pins of commercial brake lining material when compared to grey cast iron discs [15]. However, increased pin wear is inevitable due to AMC surface roughness, as well as ploughing by SiC particles on both tribosurface and counterbody. These effects play an important role in the long-term usage of AMC discs [23]. Therefore, the necessity of a stable and quick formation of a tribosurface, i.e., a fast gain of a stationary tribological system behaviour, is crucial. Previous results showed that high kinematic roughness induced by the face-turning process quickens the formation of tribosurfaces on AMC when tested against brake lining pins [17]. The results of this study confirm these findings. However, they also reveal differences in the effects on both counterbody wear and tribosurface homogeneity. The investigated surface conditions, such as surface *R*, showing kinematic roughness, and surface *E*, with an isotropic structure, both promote a quick formation of the tribosurface, but with less pin wear and a more homogeneous tribosurface for AMC condition *E*. It must be emphasised that all investigated surface conditions show nearly no disc wear during dry sliding wear against the used brake lining material (Figure 4).

The SE images in Figure 7 also confirm the interpretation about the ongoing formation and removal. They show a similar surface topography for surface *R* during all stages of the preconditioning test, while obvious changes in the appearance of the surfaces of the *F* and *E* samples occur between 2000 and 10,000 overruns. The measured surface volumes (Figure 9) confirm this by a nearly constant *V*_Surf_ level for the *R* samples between 0.30 mL/m^2^ and 0.40 mL/m^2^ that probably results from the dominating turning marks. Again, surfaces *F* and *E* show a constant drop of this value during the preconditioning test down to about 0.2 mL/m^2^. This is not only correlated with a smoother surface, but also the wear mechanism and wear rates. Since the different sample types start at different *V*_Surf_ levels (about 0.90 mL/m^2^, 0.70 mL/m^2^, and 0.50 mL/m^2^ for *R*, *F*, and *E* samples, respectively), the starting AMC surface topography obviously determines the severity of abrasion wear on the pin by the AMC surface and the system’s AR rate (the respective pin wear). Hence, surfaces *F* and *E* cause reduced wear on the pin, and thus less pin material is included in their surface topography. Furthermore, this process is slower. However, the inclusion has a higher effect on the surface smoothening than pressure in this study. It also affects the contact conditions in the tribological systems with various AMC surfaces and at different preconditioning states, i.e., the contact areas of AMC and pin material, and the contact areas of (transferred) pin material and pin material. 

The *V*_Surf_ levels and resulting contact conditions also affect the friction. The measured *µ* values for all systems vary between 0.3 and 0.4 and between 0.4 and 0.5 for high and low test pressure (i.e., 5 MPa and 2 MPa), respectively, which are suitable for braking applications [24]. The friction value variation occurs because a higher applied test load results in flattened surfaces and thus in a higher average contact area between pin and disc, as well as higher local temperature. Hence, as the normal load increases, a tribologically conditioned reaction layer under the formation of metal oxides can develop on the surface, creating low adhesion and lubricating and hence reduce the friction [12]. The low emphasis of this effect at the comparatively rough surface *R* backs this interpretation. While the *µ* values of test setups using surfaces *F* and *E* are nearly identical, the *µ* of setups with surface *R* is significantly lower. This is possibly caused by a smaller contact area (indicated by higher *V*_Surf_ values), a resulting constantly high pressure in this area, and thus a higher transfer rate of pin material to the AMC surface. Figure 8 (left column) shows that this material transfer is apparently dominant at the ridges of the turning marks in the cutting direction. At these ridges, the described tribochemical reaction can take place already at lower total test pressures. Another explanation for the smaller *µ* could be that friction-lowering debris from the pin is trapped in the turning mark valleys and thus acts as a solid lubricant. It should be added that the high AR levels (or pin wear rates), combined with the low *µ* values in systems with *R* samples, also lead to the interpretation that the test design does not measure the actual friction force, *F*_f_, but the force that is necessary to shear/cut the pin material. In this case, a comparison of the low *µ* values with the *µ* values of the *F* and *E* samples is not possible. 

The tribosurfaces were also characterised for their chemical composition by using EDX analysis, as shown in Figure 8. It was observed that the composition of a tribosurface varied from area to area, exhibiting differences in the thickness of the transferred pin material over the surface. Uyyuru et al. [16] observed this too. This is mainly due to the heterogeneous composition of the pin material, the short test duration (i.e., duration of tribological treatment), and the associated low pin wear. As a result, the probability that every phase of the pin material was worn in every area of the tribological track is very low. Figure 8 additionally demonstrates that the surface contact areas of the examined surfaces *R, F*, and *E* are completely different from one another throughout the initial and subsequent preconditioning processes. It is difficult to describe the formation of the tribosurface with Cu for the reasons given in the Section 3. Nevertheless, Ba, the well-dispersed pin-material phase, presumably present as barium sulphate, indicates that the most homogeneous transfer occurred on surface *E*, while the transfer on surfaces *R* and *F* was mainly locally concentrated at the ridges of the turning marks (*R*) or collected and condensed in the topographic holes of previously pulled out SiC_p_ in surface (*F*). Besides the elements Cu and Ba, as representative elements of pin material, oxygen mappings were created (see Figure 8). Based on the higher oxygen counts of tribosurface during EDX measurement, the presence of oxides and hydroxides can be assumed from these mappings. Tribo-oxidation may have taken place next to compositional mixing [16]. As can also be seen in Figure 8, oxygen was still detected on surface *E* despite the high copper transfer, which resulted in a lower amount of collected Al. This additionally supports the consideration that the oxidation of the transferred pin material occurred at the contact surface between the pin and the AMC disc while testing. Therefore, it can be inferred that the EDX detected/mapped oxygen is not only caused by Al_2_O_3_ due to the passivation of Al surface. 

Expressing the above in other words, even though the highest loss of pin material was observed for surface *R*, surface *E* shows the largest area of pin material transfer or tribologically conditioned reaction layer in relation to the AMC surface beneath. The minimum measured mass change (Figure 4) confirms this observation. Hence, the *E* sample exhibits the highest efficiency to form a tribological transfer layer. The protruding SiC_p_ particles, resulting from the electrolytically removed aluminium matrix on the AMC surface, in combination with the low abrasive effect of the comparatively smooth *E* sample surface, are responsible for this effect. This effect is less accentuated and also visible for the *F* sample.

In summary, surfaces *R* and *E* both showed successful behaviour with respect to the preconditioning process: On the one hand, surface *R* was characterised by a high wear rate, which led to a rapid removal of the pin material. This mainly ensured the rapid levelling of the surfaces by filling surface irregularities. Nevertheless, the surface features designated as rough surfaces often led to friction and wear results with significant standard deviations in the applied wear tests, indicating less reliable data. On the other hand, surface *E* is characterised by a very uniform transfer of pin material to the AMC surface (see Ba transfer, Figure 8), as well as constant and reliable tribological behaviour. In addition, this surface involves a higher coefficient of friction, which could be relevant for later application as a brake disc surface. However, the preconditioned surface only fully developed after a long test period (over 2000 overruns). To evaluate a possible technological application, it must also be taken into account that, during the preconditioning of the *E* sample, debris was constantly extracted that would otherwise have led to severe wear due to three-body contact (see Section 2.3).

## 5. Summary and Conclusions

In the present study, the effects of surface treatment and pin-on-disc test conditions were investigated and discussed. The aim was to speed up the formation of a tribologically conditioned surface to achieve steady state in wear and friction as fast as possible. For this purpose, three different surface types were prepared by face turning with different indexable inserts and subsequent electrolytical treatment. The so-called rough-turned (*R*), fine-turned (*F*), and electrolytically treated (*E*) surfaces were tested by dry sliding wear against common brake lining material. The main results of this study can be summarised as follows:

− At both low and high test pressures, common brake lining material against surface *R* showed the lowest coefficients of friction (≈0.3); however, it had high fluctuations. For surfaces *F* and *E*, the coefficients of friction were at higher values (between 0.4 and 0.5) and showed higher stability throughout the tests.
− The preconditioning test using surface *R* exhibited the highest level of adjustment relevelling (AR). Surface *R* demonstrated ten- and six-times higher AR rates than surfaces *F* and *E*, respectively, for tests applying both a low and high test pressure.
− The SEM images showed that pin material transfer to the surface of *R* was more pronounced at the beginning of preconditioning, but the total amount of pin material transfer to surface *E* was more significant. The lowest pin material transfer was observed for surface *F*.
− According to the surface-volume data obtained by using a novel approach (*V*_Surf_), the surface *R* showed no changes in surface volume during tests, whereas the values for surfaces *F* and *E* gradually decreased with proceeding preconditioning.

It was found that surface *R* was initially flattened and densified by pin material transfer, which was induced by severe abrasion of pin material due to the rough-turned AMC surface. Surfaces *F* and *E* showed no distinct abrasive effect, which resulted in low AR rates in comparison to surface *R*. The protruding SiC particles of the electrochemically treated surface *E*, on the other hand, promote the gradual and homogeneous transfer of pin material and the homogeneous development of a tribological layer.

According to the results of this study, further investigations are necessary to identify technologically applicable parameters for the preconditioning of AMC surfaces in tribological applications, e.g., brake discs in the automotive industry.

## Figures and Tables

**Table 1:** Generation of various surface conditions by machining and post-treatment before pin-on-disc tests.

Surface Condition of Samples	Tool Shape	Clearance Angle	Tool Radius (mm)	Feed (mm)	Cutting Speed (m/min)	Depth of Cut (mm)	Post Treatment
***R*** (rough turned)	Rhombic	7°	0.2	0.15	250	0.25	-
***F*** (fine turned)	Round	7°	3.0	0.05			-
***E*** (electrolytically treated)	Rhombic	0.5°	0.8	0.05			Plasma electrolytic

**Table 2:** Experimental setup of pin-on-disc tests.

Parameter Set	*p* (MPa)	Number of Overruns after Pressure Is Reached		
		Short	Medium	Long
Low pressure (*LP*)	2	500 overruns ≈ 65 m (in total: 245 m)	2000 overruns ≈ 265 m (in total: 445 m)	10,000 overruns ≈ 1320 m (in total: 1500 m)
High pressure (*HP*)	5			

## Data Availability

The authors confirm that the data to support the findings of this study are available within the article.

## Nomenclature

AMC	Aluminium matrix composite	
AR	Adjustment relevelling	µm
AR rate	Adjustment relevelling rate	µm/m
BSD	Backscatter diffraction	
*E*	Electrolytically treated	
EDX	Energy dispersive X-ray spectroscopy	
*F*	Fine turned	
FAST	Field assisted sintering technique	
*F*_N_	Normal load	N
*F*_f_	Friction load	N
*HP*	High pressure	
*LP*	Low pressure	
MRC	Material ratio curve	
*p*	Pressure	MPa
*R*	Rough turned	
*R* ^2^	Coefficient of determination	
SE	Secondary electrons	
SEM	Scanning electron microscope	
SiC_p_	Particulate silicon carbide	
*Spk*	Reduced peak height	µm
*Svk*	Reduced valley height	µm
*t*	Time	s
*v*_c_	Cutting speed	m/min
*v*_f_	Feed velocity	mm/min
*V*_Surf_	Surface volume	mL/m^2^
*µ*	Friction coefficient	
*ϑ*	Temperature	°C
